# Results of a CCPR Intercomparison of Spectral Irradiance Measurements by National Laboratories

**DOI:** 10.6028/jres.096.043

**Published:** 1991

**Authors:** James H. Walker, Robert D. Saunders, John K. Jackson, Klaus D. Mielenz

**Affiliations:** National Institute of Standards and Technology, Gaithersburg, MD 20899, USA

**Keywords:** CCPR, intercomparison, national laboratories, radiometry, spectral irradiance, tungsten-halogen lamps

## Abstract

An intercomparison of spectral irradiance measurements by 12 national laboratories has been carried out between 1987 and 1990. The intercomparison was conducted under the auspices of the Comité Consultatif de Photometrie et Radiometrie (CCPR) of the Comité International des Poids et Mesures, and the National Institute of Standards and Technology (NIST) served as the pilot laboratory. The spectral range of the intercomparison was 250 to 2400 nm and the transfer standards used were commercial tungsten-halogen lamps of two types. The world-wide consistency of the results (one standard deviation) was on the order of 1% in the visible spectral region and 2 to 4% in the ultraviolet and infrared portions of the spectrum. The intercomparison revealed no statistically significant differences between spectral-irradiance scales based on blackbody physics and absolute detector radiometry.

## 1. Overview

At the September 1986 Session of the Comité Consultatif de Photometrie et Radiometrie (CCPR) of the Comité International des Poids et Mesures [1], the delegates of the National Institute of Standards and Technology (NIST) proposed an intercomparison of the spectral-irradiance scales maintained and disseminated by national standards laboratories throughout the world. The proposal received wide support and the Radiometric Physics Division of NIST was appointed the pilot laboratory for the intercomparison. It was agreed that the intercomparison would cover the spectral region from 250 to 2400 nm, and that the following schedule would be adhered to:

1987/88 – Invitation to participants, procurement of intercomparison lamps, lamp mounts, and alignment jigs.
1989/90 – Initial calibration of a set of three lamps by each participant, calibration of all lamps by NIST, and repeat calibration of each set of lamps by the participants.

The intercomparison was to be “blind,” in that the NIST results would not be revealed to the participants until their repeat calibrations had been completed.

A preliminary NIST report of the intercomparison was presented at the September 1990 Session of the CCPR [2]. The committee decided to allow no more “fine tuning” of data, and appointed a working party convened by NIST to prepare a final report in which the results of the intercomparison are presented in terms of the differences,

$$r=\frac{\text{Average of}\;“\text{before}”\;\text{and}\;“\text{after}”\;\text{measurements by participants}}{\text{NIST measurement}}-1.$$ (1)

This value was multiplied by 100 to obtain the percent difference from NIST. It was noted that the quotients in Eq. (1) are reciprocal to the equivalent quotients used in the 1975 spectral-irradiance intercomparison coordinated by the Electrotechnical Laboratory (ETL) of Japan [3] and that in the latter intercomparison the final data were adjusted to show the differences of each participant (including ETL) from a “world mean” of zero.

## 2. Participants

Twelve national laboratories participated in the intercomparison.^1^ They are listed below, with the names of the principal investigators assigned to the intercomparison given in parentheses.

CSIRO: Commonwealth Scientific and Industrial Research Organization, Division of Applied Physics, Lindfield, Australia (J. L. Gardner).
ETL: Electrotechnical Laboratory, Ibaraki, Japan (M. Nishi).
INM: Institut National de Metrologie du Conservatoire National des Arts et Niederes, Paris, France (J. Bastie).
IOM: Instituto de Optica Daza de Valdes, Madrid, Spain (A. Corrons).
NIM: National Institute of Metrology, Beijing, People’s Republic of China (Chen Xiaju).
NIST: National Institute of Standards and Technology, Gaithersburg, MD, USA (J. H. Walker).
NPL: National Physical Laboratory, Teddington, Middlesex, UK (J. R. Moore).
DPT: Division of Production Technology, CSIR, Pretoria, South Africa (F. Hengstberger).
NRC: National Research Council, Ottawa, Canada (L. P. Boivin).
OMH: National Office of Measures, Budapest, Hungary (G. Deszi).
PTB: Physikalisch-Technische Bundesanstalt, Braunschweig, Federal Republic of Germany (J. Metzdorf)
VNIIOFI: All-Union Research Institute of Optical and Physical Measurements, Moscow, U.S.S.R. (V. I. Sapritsky).

## 3. Lamps

The lamp originally chosen for the intercomparison was a 770 W (14 A at 55 V dc) tungsten-bromine lamp that had been developed jointly by the National Physical Laboratory (NPL) and the General Electric Company (GEC) of the United Kingdom for use both as a standard of spectral irradiance and as a standard of illuminance at a correlated color temperature of 3000 K [4]).^2^ The filament assembly of the lamp, consisting of six vertical tungsten coils arranged in a 16 × 24 mm plane, is enclosed in a fused-silica envelope filled with 304 kPa (3 atm) of nitrogen (equivalent to 1013 kPa (10 atm) when the lamp is operating). The lamp is equipped with a commercial 22 mm bi-pin base and is operated base down. Initial testing at the NPL had shown that the lamp required aging for 300 to 400 h on dc in order to achieve a stability in illuminance of no worse than 0.5% per 100 h of use. The lamp obeyed the inverse-square law for working distances greater than 200 mm. Its uniformity of field was found to be better than ±0.5% over an angular range of 5° subtended at the lamp in the direction of a horizontal axis through the center of, and perpendicular to, the filament plane.

NPL had agreed to select and deliver three of these NPL/GEC lamps for each participating laboratory. This was achieved in mid-1988, but unfortunately some of these lamps failed during the first round of measurements and replacements could no longer be obtained from GEC. In order to remedy this difficulty, NIST supplied several of its routinely issued spectral-irradiance standard lamps (General Electric Company (USA) FEL lamps) to those laboratories that had lost NPL/GEC lamps or wished to include the NIST/FEL lamp for other reasons.

A detailed description of the NIST/FEL lamp may be found in Ref. [5]. The lamp, rated at 1000 W, is a clear quartz envelope, tungsten-halogen lamp with a cylindrical coiled-coil filament of 8 mm diameter and 24 mm height. The lamp is modified to a 22 mm bi-pin base and is operated base down. The lamps are annealed at 120 V dc for 40 h (13% of its rated life), and then burnt in for 24 h under normal operating conditions (7.7 to 8.0 A at 106 to 112 V dc) to test their stability. Only lamps with changes less than 0.5% in 24 h at 655 nm are accepted. All lamps are tested for irradiance uniformity over a +1° range of rotation and tilt, and lamps exhibiting changes greater than 1% are rejected.

**Figure 1:**
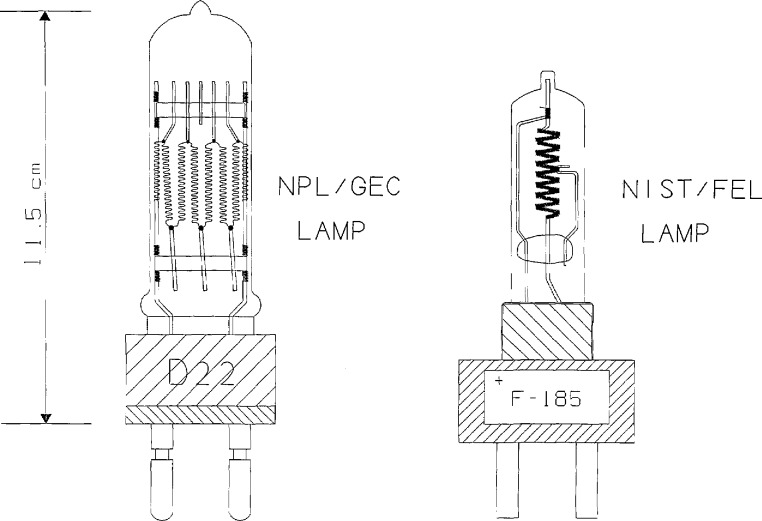
Lamps used in the spectral irradiance intercomparison.

**Figure 2:**
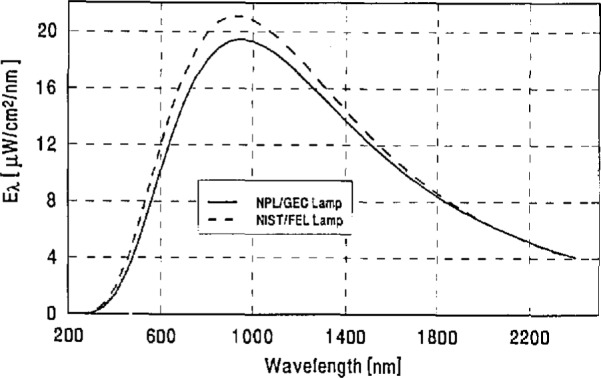
Average spectral irradiances of NPL/GEC and NIST/FEL lamps at a distance of SO cm from lamp.

Schematic drawings of the NPL/GEC and NIST/FEL lamps are shown in Fig. 1. NIST supplied alignment jigs and detailed alignment instructions for each lamp type. The lamps were to be mounted vertically, base down, and measured at a distance of 500.0 mm between a specified reference plane and the aperture of the receiving instrument. The average spectral irradiances (in μ w/cm^2^/nm) produced by the lamps under these conditions are plotted in Fig. 2. From these data it was estimated that the approximate correlated color temperatures of the lamps were 2979 K for the NPL/GEC lamps and 3075 K for the NIST/FEL lamps.

The final count of lamps used in the intercomparison was 25 NPL/GEC lamps and 6 NIST/FEL lamps. Most participants contributed data for three lamps. Two laboratories (ETL, VNIIOFI) contributed data for two lamps.

## 4. Measurement Procedures at Participating Laboratories

All lamps were operated at a constant current and their voltage was monitored. All the NPL/GEC lamps were operated at 14.000 A with a nominal voltage of about 55 V. The NIST/FEL lamps were operated at either 7.700, 7.800, or 7.900 A with nominal voltages of 106 to 109 V.

The information provided by the participating laboratories on the measurement methods used by them may be found in Appendix A of this paper. This information is summarized in Table 1, and the following points are worth emphasizing.

- Six laboratories (ETL, INM, NIM, NIST, PTB, VNIIOFI) had independently realized their spectral-irradiance scales by blackbody physics. All of these scales were stated to be consistent with the International Temperature Scale of 1990 [6] that was adopted during the intercomparison. Two scales (IOM, DPT) and the infrared portion of another (NRC from 700 nm upwards) had been independently realized by electrically calibrated radiometers. Two laboratories (CSIRO, NPL) reported that the 350 to 800 nm portions of their scales were based on relative spectral distributions derived from blackbodies, with absolute values assigned by photometric measurements. The NPL ultraviolet scale below 370 nm was based on synchrotron radiation. The NPL infrared scale from 900 nm upwards was based on provisional calculations using published data for the emissivity of tungsten because their new infrared scale was not completed in time for the intercomparison. The remaining scales (CSIRO below 350 nm and above 800 nm, NRC from 300 to 700 nm, OMH) were based on transfer standards provided by other national laboratories.
- Most laboratories contributed data over significant portions of the uv, visible, and near ir, but only five laboratories (CSIRO, ETL, NIM, NIST, PTB) covered the entire 250 to 2400 nm intercomparison range.
- All laboratories used medium-sized (0.25 to 1 m focal length) spectroradiometers. All used S-20 type photomultiplier tubes for measurements in the uv and visible. The detectors used in the infrared were Si and Ge photodiodes or PbS photoconductive cells, and several laboratories used two of these detectors.
- All laboratories performed the measurements using routine calibration methods and procedures. However, most laboratories performed the measurements using highly qualified staff and special care. Tests of wavelength accuracy, stray radiant energy, detector linearity and ambient temperature were performed as a routine matter.

All laboratories were requested to provide one standard deviation estimates of their measurement uncertainty, with random and systematic errors added in quadrature. These estimated uncertainties are listed in Table 2.

## 5. Measurement Procedure at NIST

**Figure 3:**
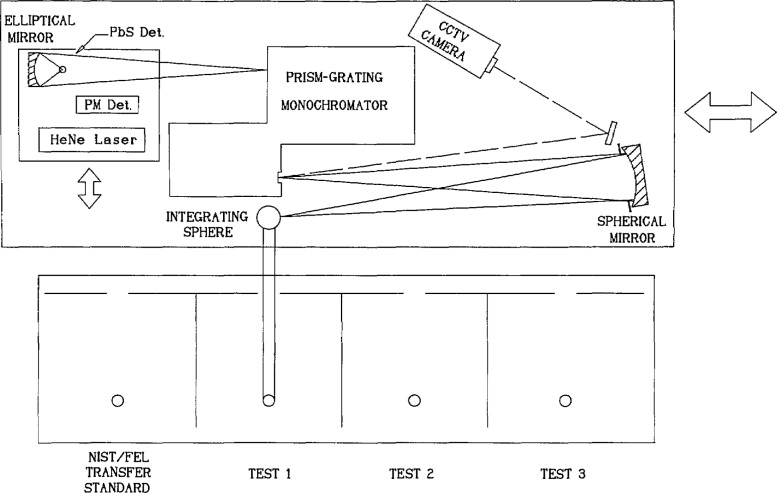
NIST spectral irradiance measurement equipment.

The spectral irradiance measurements performed at NIST were made relative to the 1990 NIST scales of thermal radiometry [7] and are fully consistent with the ITS-90. Before and after spectral irradiance measurements on all intercomparison lamps were performed, once in September/October 1989 and again in April/May 1990, the NIST spectral irradiance scale was realized by calibrating a group of nine NIST/FEL transfer standards against a gold-point blackbody standard, using the measurement procedures described in Refs. [5] and [7]. All NIST measurements contributed to the intercomparison were derived by linear interpolations between these two scale realizations. The measurement setup used to compare the participants’ intercomparison lamps to the NIST transfer standards is shown in Fig. 3. Each intercomparison lamp was measured against each of four NIST/FEL transfer standards, and the four spectral-irradiance values thus obtained for each lamp were averaged. The total burning time at NIST was on the order of 20 to 25 h for each lamp. A more detailed description of the NIST measurements may be found in Appendix A.

## 6. Data Analysis

After the completion of all measurements, the following data analysis was carried out by NIST staff.

- The percent differences from NIST defined by Eq. (1) were computed as a function of wavelength for both the round-one and round-two spectral irradiance values reported by the participating laboratories for each intercomparison lamp.
- The differences between these round-one and round-two differences from NIST were compared to the lamp voltage readings recorded for each lamp by the participants and by NIST. This comparison revealed a few cases in which unduly large discrepancies between the participants’ round-one and round-two measurements appeared to be caused by bistable behavior of a lamp. Upon notification of these findings, five laboratories requested that the measurements of one of their lamps be excluded from the data analysis. The average absolute differences between the round-one and round-two data for the remaining lamps were small (ranging from 1.4% at 250 nm through 0.5% at 600 nm to 1% at 2400 nm) and were not included in the error analysis.
- The averages of the round-one and round-two percent differences from NIST were computed for each lamp, and the average of these differences of all the lamps measured by each participating laboratory was computed to serve as the “grand mean” for each laboratory. A sample of the data analysis for one laboratory is shown in Appendix B.
- As an independent measure for judging the statistical significance of the grand-mean differences, the quadrature combination of the one standard deviation uncertainties quoted by NIST and each laboratory was computed.

Appendix C contains a plot for each laboratory showing its grand mean percent difference from NIST and the combined Lab/NIST one standard deviation uncertainty.

## 7. Results and Discussion

**Figure 4:**
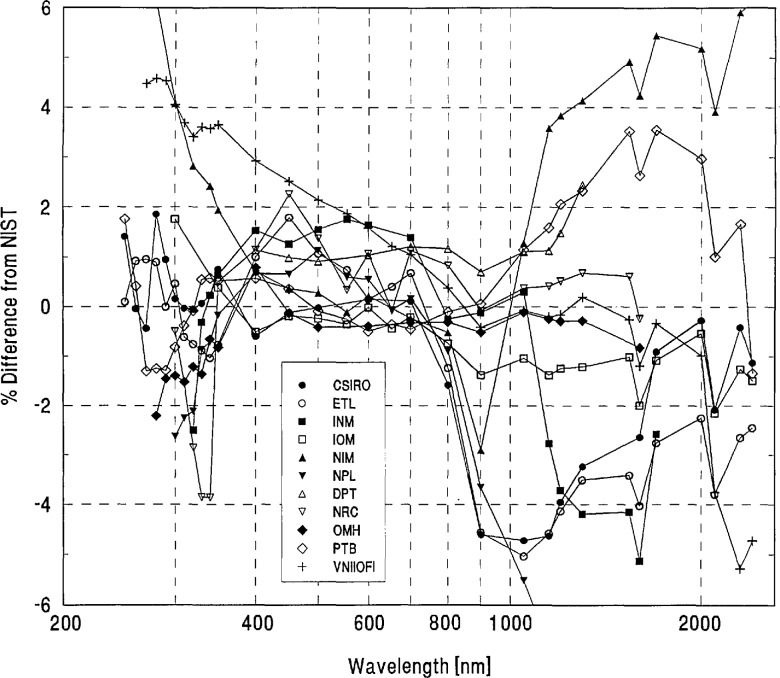
Grand-mean percent differences from NIST of all participants’ spectral-irradiance measurements.

Table 2 shows the grand-mean percent differences from NIST and the one standard deviation uncertainties of each laboratory’s measurements. A plot of the grand-mean differences versus wavelength is shown in Fig. 4. Differences greater than ±6% are not shown on this plot, but can be found in Table 2.

As a visual aid in relating the results obtained for each laboratory to their estimated uncertainties, every grand-mean difference that exceeds 1.1 times^3^ the combined laboratory/NIST uncertainty associated with it (as defined in Sec. 6(d), above) has been highlighted in Table 2. The number of measurements thus identified is:

ultraviolet region (250 to 350 nm) 16 of 75 measurements (21%)
visible region (400 to 800 nm) 15 of 80 measurements (19%)
infrared region (900 to 2400 nm) 48 of 102 measurements (47%)

This result should, however, be viewed with caution because it depends on the uncertainty estimates provided by the participating laboratories and because these estimates vary considerably among laboratories. We noted that the participants’ uncertainty estimates for many of the highlighted measurements in Table 2 are quite low, and that there are several instances in which grand-mean differences of similar magnitude but larger estimated uncertainties survived the “highlighting” criterion used in Table 2. In this context it may be of interest to compare the uncertainties estimated by each laboratory to the average of the estimates provided by all laboratories. This average, excluding the uncertainty of measurements which deviate significantly from the world mean (NIM in the 250 to 280 nm region and NPL in the 900 to 2400 nm region) is shown in Table 3 as a function of wavelength, and hence it may be seen that the participants’ uncertainty estimates are lower than average in the case of 54 of the 79 highlighted measurements in Table 2. This appears to indicate that the uncertainties assigned by some national laboratories may be too small, at least at the one standard deviation level.

As a measure of the world-wide consistency of spectral-irradiance measurements we calculated both the average and the standard deviation of the grand-mean differences from NIST, again excluding the measurements which deviate significantly from the world mean (NIM in the 250 to 280 nm region and NPL in the 900 to 2400 nm region). The results of this calculation are also given in Table 3 and show that the world-average difference from NIST is within the world-average uncertainty estimate for every intercomparison wavelength and exceeds its own standard deviation at one wavelength only (250 nm). However, the standard deviation of the world-average difference from NIST is within the world-average uncertainty estimate only in the visible region, but exceeds it for several ultraviolet wavelengths and for every infrared wavelength from 900 nm upwards. Using the standard deviation of the world average as a measure, we estimate the consistency of the national scales and the intercomparison measurements to be on the order of 1% in the visible region (400 to 800 nm) and on the order of 2 to 4% in the ultraviolet and infrared regions. The overall spread of results is, of course, greater and may be inferred from Table 2.

Figure 4 and Table 3 show that the spectral-irradiance measurements performed by NIST assigned slightly lower values than the world average throughout the visible region and at several ultraviolet wavelengths, and tend to be slightly higher in the infrared region. In particular, Fig. 4 shows that dips on the order of 1% appear in most participants’ differences from NIST at 1600 and 2100 nm. These dips may be caused by variations in the NIST scale which are within NIST’s uncertainty estimates.

**Figure 5:**
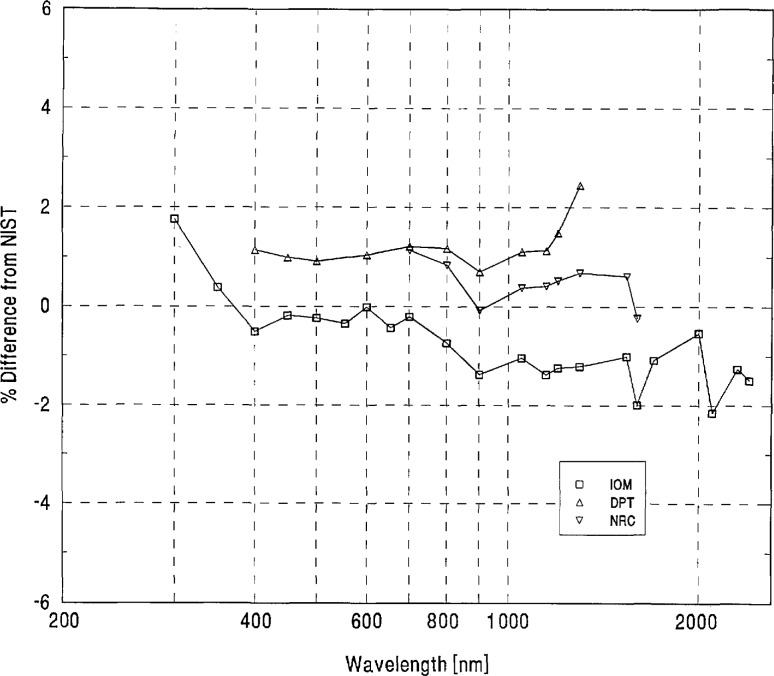
Grand-mean percent differences from NIST of participants with detector-based scales.

We looked for systematic differences between spectral-irradiance scales based on different physical principles. As noted in Sec. 4(a), the NIST scale and a large number of the others are based on blackbodies. As may be seen in Fig. 5, the three scales based on absolute detector radiometry (IOM, DPT, and NRC above 700 nm) show no significant differences from NIST or from the world average and were therefore judged to be consistent with blackbody scales. However, the three detector based scales appear to have better agreement in the irregion than the non-detector based scales. The only other measurements not based on black-body physics (the synchrotron-based NPL measurements below 370 nm) appear to have yielded somewhat lower values than NIST near 300 nm.

For comparison purposes, we have included on the right-hand side of Table 3 the standard deviations of the world averages obtained in the 1975 spectral-irradiance intercomparison conducted by ETL [3].^4^ This shows that the results of the present intercomparison are not too different, except that more laboratories participated and that a greater spectral region was covered.

## Figures and Tables

**Figure 6:**
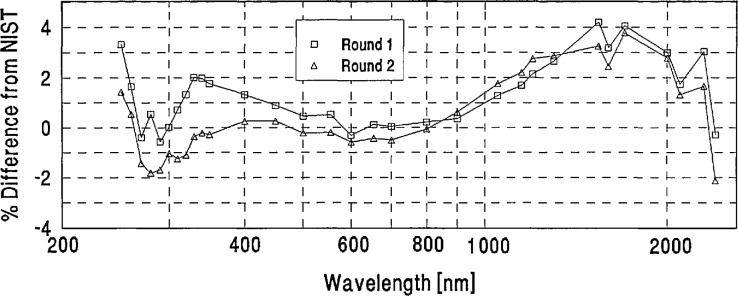
PTB Lamp E9 percent difference from NIST.

**Figure 7:**
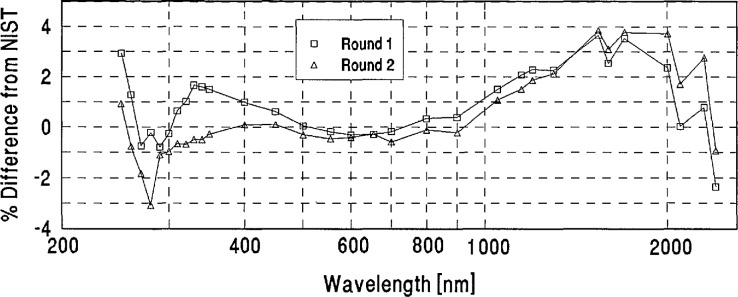
PTB lamp E10 percent difference from NIST.

**Figure 8:**
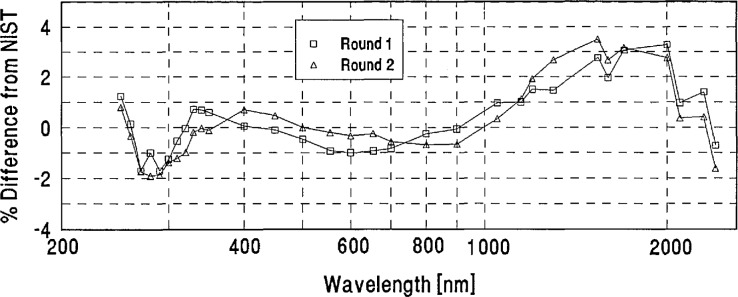
PTB lamp F28 percent difference from NIST.

**Figure 9:**
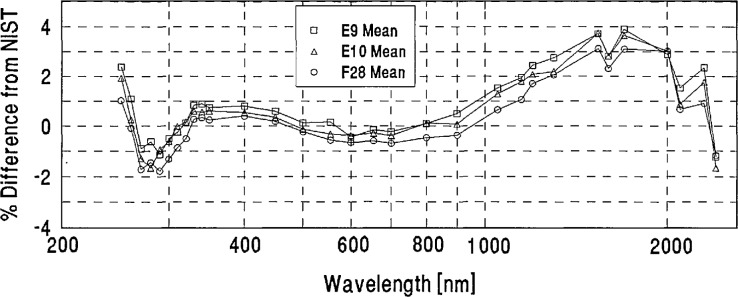
PTB percent difference from NIST.

**Figure 10:**
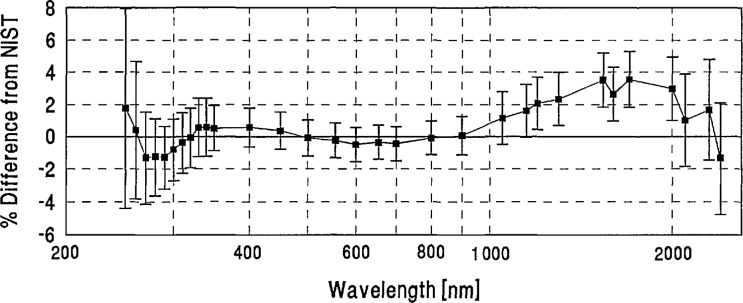
PTB grand-mean percent difference from NIST and combined PTB/NIST uncertainty.

**Figure 11:**
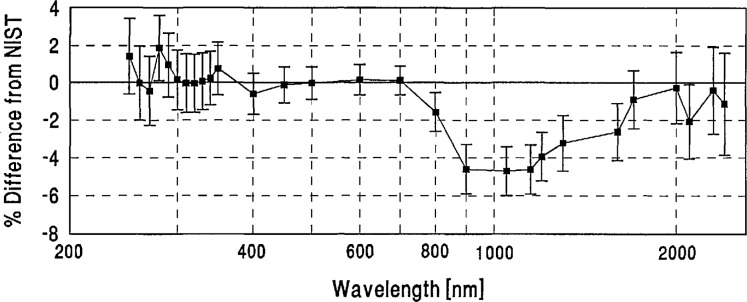
CSIRO grand-mean percent difference from NIST and combined CSIRO/NIST uncertainty.

**Figure 12:**
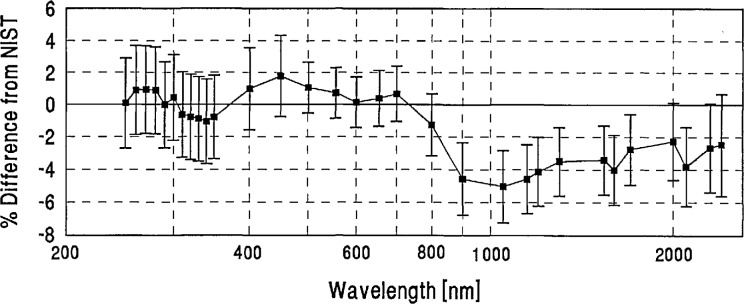
ETL grand-mean percent difference from NIST and combined ETL/NIST uncertainty.

**Figure 13:**
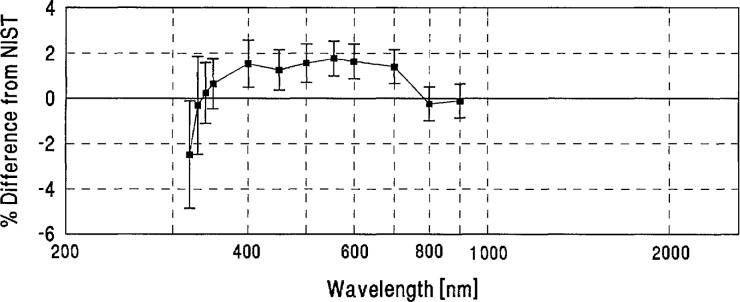
INM grand-mean percent difference from NIST and combined INM/NIST uncertainty.

**Figure 14:**
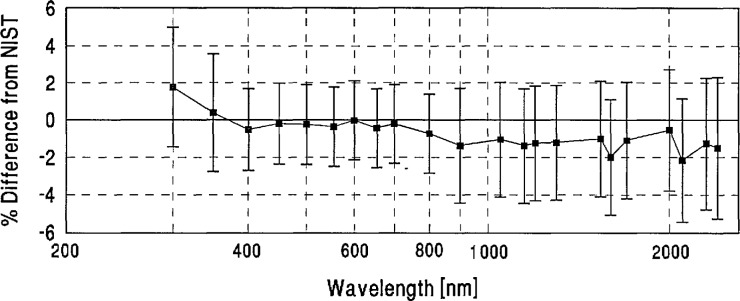
IOM grand-mean percent difference from NIST and combined IOM/NIST uncertainty.

**Figure 15:**
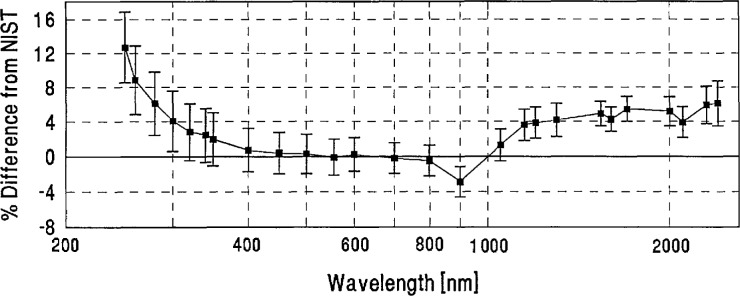
NIM grand-mean percent difference from NIST and combined NIM/NIST uncertainty.

**Figure 16:**
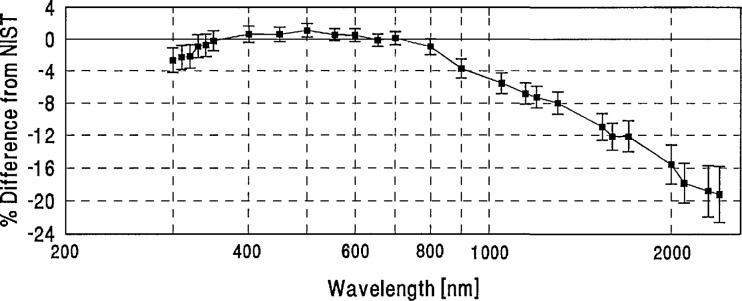
NPL grand-mean percent difference from NIST and combined NPL/NIST uncertainty.

**Figure 17:**
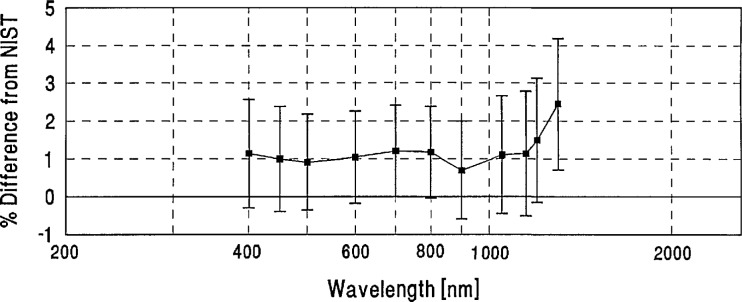
DPT grand-mean percent difference from NIST and combined DPT/NIST uncertainty.

**Figure 18:**
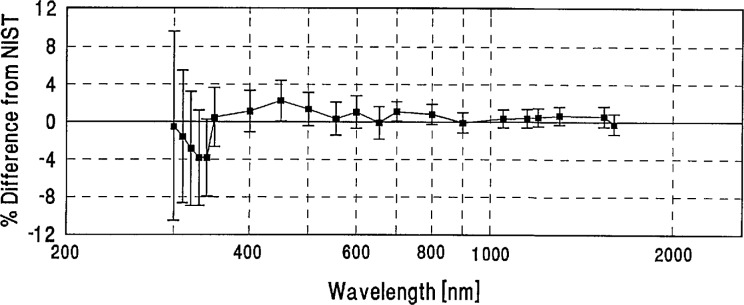
NRC grand-mean percent difference from NIST and combined NRC/NIST uncertainty.

**Figure 19:**
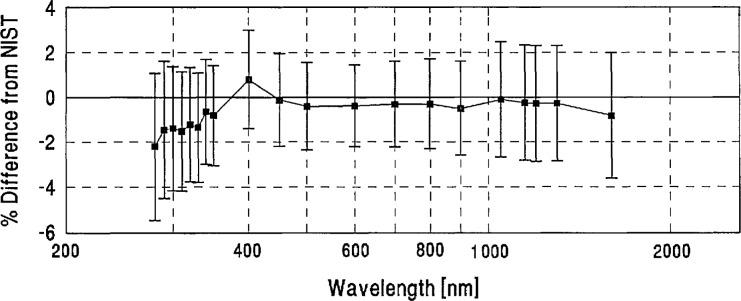
OMH grand-mean percent difference from NIST and combined OMH/NIST uncertainty.

**Figure 20:**
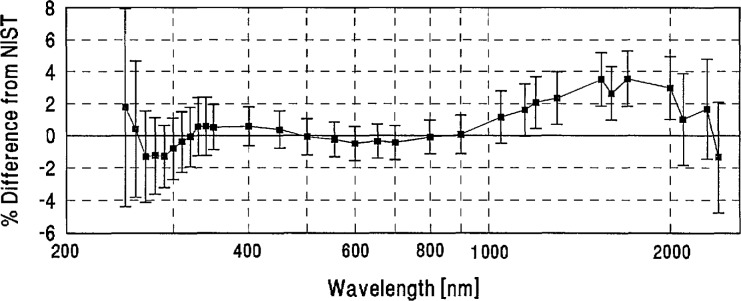
PTB grand-mean percent difference from NIST and combined PTB/NIST uncertainty.

**Figure 21:**
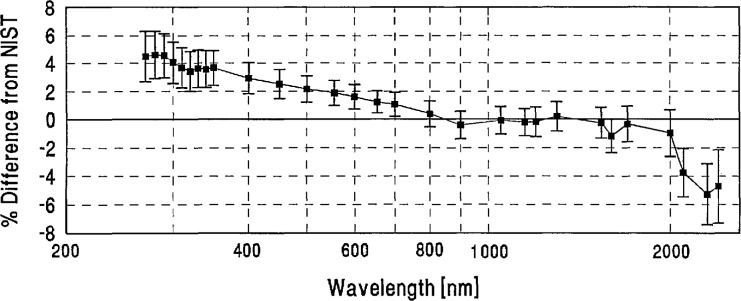
VNIIOFI grand-mean percent difference from NIST and combined VNIIOFI/NIST uncertainty.

**Table 1:** Summary of measurement conditions at participants’ laboratories

	CSIRO	ETL	INM	IOM	NIM	NIST	NPL	DPT	NRC	OMH	PTB	VNIIOFI
Sp Irr Scale												
Primaiy Std	AR/BB/CL^a^	BB	BB	AR	BB	BB	AR/BB	AR	AR/CL^c^	CL^d^	BB	BB
Trsf Std	TH	QB	CL^b^	TH	TH	TH	TH	None	None/TH	TH	DXW.FEL	BB

Lamps												
NPL/GEC	E14.E36	E20,(F15),F32	E25	E8,F24,F25	F6,F7,F8		(E23),E24	E15.E16.E17	E2.E3	E31,E32 F2	E9,E10,F28	D30,(D31),D32
NIST/FEL	(H146)^g^, H148		F295_1_F296,(F297)				F287.F291		F176			
Comments												

Monochromator	double^h^	double	single	single^e^	double	double	double^f^	double	single^i^	double	double	double
Make	McPherson	JASCO	Jobin-Yvon	Jarrell-Ash	DMR	Cary	Jobin-Yvon	Jobin-Yvon	McPherson	Hilger-Watts	Cary	Jobin-Yvon
Type	grating	prism/grating	grating	grating	prism	prism/grating	grating	grating	grating	grating	prism/grating	grating
focal length	0.5 m	0.4 m	0.6 m	0.75 m	0.25 m	0.4 m	0.6 m	0.6 m	1m	0.3m	0.4 m	0.64 m
*f*-number	7	6.8	5.6	6.5	10	8	5.7	5.7	8.7	6	8	5.7

UV-VIS Range	250–700	250–700	300–1000	350–800	250–800	250–800	300–800	400–700	300–700	280–900	250–800	270–800
BW @250 nm	2nm	4 nm			3 nm	4.6 nm					6 nm	
400 nm	2	1.5	2nm	1.8 nm	1.6	7.4	2 nm	2.2 nm	2 nm	4 nm	0.4	1.2 nm
700 nm	2	1.5	2	2.1	1.8	8.4	2	2.2	2	2.6	0.8	2.8
Detector	9558QB	R374	R546	9558QB	9659QB	9659Q	9558QA	R928	R758	9558QB	9558QB	PMT (two)^j^
Frequency	dc	1kHz	dc	dc	800Hz	dc	dc	dc	dc	268Hz	dc	dc
Int time	3–10 s	300 ms	40s	1s	1s	3–10 s	1 s	10 s	2.1 s	1–30 s	4s	1s

IR Range	700–2400	800–2400	1100–1700	800–2400	750–2500	900–2400	900–2400	700–1300	700–1600	1050–1600	800–2400	900–2400
BW @1050 nm	2 nm	10 nm	2 nm	7.2 nm	43 nm	7.2 nm	2 nm	1.8 nm		10.4 nm	2.4 nm	*2.5* nm
1600 nm	8	10	4	14	65	6.8	2			10.4	11	5.2
2400 nm	8	10		14.4	88	5.2	4				9	4.8
Detector 1	Si UV800B	Si S2592	Si 10D	PbS	PbS	PbS	PbS	SiUV4448	ECR & filter	Si S1337	Ge E70	PbS
Frequency	dc	1kHz	dc	141 Hz	800 Hz	78 Hz	200 Hz	dc	dc	268 Hz	133 Hz	510 Hz
Int time	3–10 s	300 ms	40 s	0.3 s	1 s	8–40 s	2.5 s	10 s	30 s	1–30 s	10 s	1 s
Detector 2	PbS	PbS P1026	Ge	n/a	n/a	n/a	n/a	Ge J16	n/a	PbS P2532	PbS H340	n/a
Frequency	300 Hz	1 kHz	10 Hz					32 Hz		268 Hz	133 Hz	
Int time	3–10 s	300 ms	40 s					10 s		1–30 s	25 s	

Tests Performed												
Wvlgth Ace	Hg lines	Hg and D2 lines	Hg lines and HeNe laser	Hg lines; Kr laser	Hg lines	Hg, Th lines	Hg, Cd lines <0.1 nm uncert	Hg and Cd lines	Hg, Cd Unes	Hg, Cd and Ne lines	Hg lines	Hg lines
Scat Light	cut-off *f*	cut-off *f*	bandpass *f*	cut-off *f*		no effect	cut-off *f*	backgrd sub	baffles	cut-off *f*	no effect	cut-off *f*
Det Lin	mult ap	not checked	light add	dbl beam	mult ap	dbl beam	dbl beam	dbl beam	checked 1979	dbl ap	1/*r*^2^	mult ap
Amb Temp	21±.5	23±1	measured	measured	measured	23–25	22±1	22–24	24–26	23±1	23±1	measured

a 350–800 nm: tungsten tube cavity radiator at 2700 K and V(lambda) radiometer. 250–350 nm and 800–2400 nm: lamps from ETL.

b Spectral radiance lamps and luminous intensity lamps.

c 300–700 nm: lamps based on 1975 CIE intercompanson. 700–1600 nm: ECR.

d TH lamps from NIST.

e Used two monochromators: Jarrell-Ash used from 350–800 nm; Jobin-Yvon (double grating) used from 800–2400 nm with 0.25 m focal length and 35*f*-number.

f Used two monochromators: Jobin-Yvon (double Czerny-Turner) used from 300–2400 nm; Hilger Uvisir (double prism Littrow) used from 300–800 nm.

g Parentheses around a lamp number indicates an excluded lamp.

h Double in UV-VIS. Single with prism predisperser in IR.

i Has prism predisperser.

j Two Soviet made PMTs: one for 280–600 nm, one for 600–900 nm.

Key to symbols in table:
ARAbsolute RadiometerQBQuartz Bromine lampsSiSxxxx Hamamatsu Sxxxx silicon photodiodeBBBlackbodyGe J16Judson J16 germanium photodiodeSi UVxxxxEG&G UVxxxx silicon photodiodeCLCalibrated lampsRxxxHamamatsu Rxxx photomultiplier tubeGeE70 Electro Optical Systems GLN-05O/E70 germanium photodiodeDXDXW type lampsxxxxQBEMI xxxxQB photomultiplier tubePbSH340 Santa Barbara Research Center H340 PbS-ITO detectorFELFEL type lampsxxxxQEMI xxxxQ photomultiplier tubePbSPxxxx Hamamatsu Pxxxx PbS photoconductive detectorTHTungsten Halogen lampsSi 10DUDT PIN 10D silicon photodiode

**Table 2:** Grand-mean percent differences^a^ from NIST and estimated one standard deviation uncertainties of participants’ measurements

NIST		CSIRO		ETL		INM		IOM		NIM		NPL		DPT		NRC		OMH		PTB		VNIIOFI	
WL (nm)	Unc (%)	Grand mean	Unc (%)	Grand mean	Unc (%)	Grand mean	Unc (%)	Grand mean	Unc (%)	Grand mean	Unc (%)	Grand mean	Unc (%)	Grand mean	Unc (%)	Grand mean	Unc (%)	Grand mean	Unc (96)	Grand mean	Unc (%)	Grand mean	Unc (%)
250	1.48	1.42	1.4	0.10	2.4					*12.69*^b^	3.9									1.77	6		
260	1.40	−0.04	1.4	0.92	2.4					*8.86*	3.8									0.42	4		
270	1.34	−0.44	1.3	0.96	2.4															−1.31	2.5	*4.47*	1.2
280	1.28	1.86	1.2	0.90	2.4					*6.10*	3.5							−2.21	3.0	−1.25	2	*4.58*	1.1
290	1.22	0.95	1.2	0.00	2.4													−1.46	2.8	−1.29	1.5	*4.53*	1.0
300	1.17	0.16	1.1	0.46	2.4			1.76	3	*4.07*	3.3	−2.63	1			−0.49	10	−1.40	2.5	−0.82	1.5	*4.05*	0.9
310	1.13	−0.03	1.1	−0.62	2.4							−2.25	1			−1.57	7	−1.52	2.4	−0.39	1.5	*3.68*	0.9
320	1.09	−0.05	1.1	−0.76	2.4	−2.50	2.1			*2.82*	3.1	−2.12	1			−2.85	6	−1.22	2.3	−0.08	1.5	*3.41*	0.9
330	1.06	0.07	1.1	−0.89	2.4	−0.31	1.9					−0.86	1			−3.86	5	−1.37	2.2	0.55	1.5	*3.60*	0.8
340	1.03	0.23	1.0	−1.04	2.4	0.23	0.8			2.43	2.9	−0.71	1			−356	4	−0.66	2.1	0.58	1.5	*3.57*	0.8
350	1.01	0.76	1.0	−0.77	2.4	0.64	0.4	0.39	3	1.94	2.9	−0.17	0.8			0.50	3	−0.82	2.0	0.52	1	*3.65*	0.7
400	0.92	−0.60	0.6	1.00	2.4	*1.53*	0.5	−0.51	2	0.71	2.3	0.67	0.5	1.14	1.1	1.15	2	0.79	2.0	0.57	0.8	*2.92*	0.6
450	0.84	−0.12	0.5	1.79	2.4	*1.26*	0.3	−0.18	2	0.37	2.2	0.66	0.4	0.99	1.1	2.26	2	−0.13	1.9	0.36	0.8	*2.52*	0.6
500	0.78	−0.02	0.4	1.07	1.4	*1.56*	0.3	−0.24	2	0.28	2.1	*1.14*	0.4	0.91	1	1.37	1.6	−0.42	1.8	−0.09	0.8	*2.14*	0.5
555	0.73			0.75	1.4	*1.76*	0.2	−0.35	2	−0.11	1.9	0.60	0.2			0.34	1.6			−0.25	0.8	*1.87*	0.5
600	0.70	0.15	0.4	0.16	1.4	*1.64*	0.3	−0.01	2	0.22	1.8	0.55	0.4	1.04	1	1.07	1.6	−0.39	1.7	−0.50	0.8	*1.59*	0.5
654.6	0.68			0.40	1.6			−0.43	2			−0.06	0.4			−0.07	1.6			−0.35	0.8	*1.22*	0.4
700	0.67	0.12	0.4	0.69	1.6	*1.40*	0.3	−0.21	2	−0.23	1.6	0.17	0.5	1.21	1	1.14	0.8	−0.32	1.8	−0.45	0.8	*1.06*	0.5
800	0.67	*−1.58*	0.8	−1.24	1.8	−0.25	0.3	−0.74	2	−0.51	1.6	−0.90	0.8	1.17	1	0.83	0.8	−0.31	1.9	−0.10	0.8	0.38	0.6
900	0.68	*−4.60*	1.1	*−4.56*	2.1	−0.12	0.3	−1.38	3	−2.90	1.6	*−3.65*	1	0.70	1.1	−0.08	0.8	−0.52	2.0	0.06	1	−0.42	0.7
1050	0.68	*−4.72*	1.1	*−5.04*	2.1			−1.04	3	1.28	1.7	*−5.52*	1.1	1.11	1.4	0.38	0.7	−0.11	2.5	1.15	1.5	−0.07	0.7
1150	0.67	*−4.63*	1.1	*−4.58*	2.0			−1.38	3	*3.59*	1.7	*−6.81*	1.2	1.13	1.5	0.41	0.7	−0.24	2.5	1.59	1.5	−0.20	0.7
1200	0.67	*−3.95*	1.1	*−4.13*	2.0			−1.24	3	*3.84*	1.7	*−7.23*	1.2	1.49	1.5	0.52	0.7	−0.29	2.5	*2.06*	1.5	−0.16	0.8
1300	0.67	*−3.23*	1.3	*−3.50*	2.0			−1.21	3	*4.13*	1.8	*−7.99*	1.2	*2.45*	1.6	0.68	0.7	−0.28	2.5	*2.33*	1.5	0.19	0.8
1540	0.75			*−3.41*	2.0			−1.01	3	*4.92*	1.2	*−10.9*	1.5			0.61	0.8			*3.52*	1.5	−0.26	0.8
1600	0.77	*−2.63*	1.3	*−4.02*	2.0			−1.99	3	*4.24*	1.2	*−12.2*	1.5			−0.24	0.8	−0.82	2.7	*2.64*	1.5	−1.19	0.9
1700	0.87	−0.91	1.3	−2.75	2.0			−1.08	3	*5.45*	1.2	*−12.1*	1.8							*3.55*	1.5	−0.33	0.9
2000	1.30	−0.27	1.4	−225	2.0			−0.54	3	*5.19*	1.1	*−15.6*	2							*2.97*	1.5	−0.98	1.0
2100	1.39	−2.08	1.4	−3.80	2.0			−2.15	3	*3.91*	1.1	*−17.8*	2							1.01	2.5	*−3.76*	1.0
2300	1.86	−0.42	1.4	−2.65	2.0			−1.26	3	*5.92*	1.2	*−18.8*	2.5							1.66	2.5	*−5.28*	1.1
2400	2.34	−1.13	1.4	−2.45	2.1			−1.49	3	*6.14*	1.2	*−19.2*	2.5							−1.34	2.5	*−4.73*	1.1

a The average of the differences of all the lamps for each laboratory.

b *Italics* indicates the grand-mean difference exceeds 1.1 times the combined Laboratory/NIST uncertainty associated with it.

**Table 3:** World averages of one standard deviation uncertainty estimates, percent differences from NIST, and comparison with 1975 ETL intercomparison

WL (nm)	World average of the estimated uncertainties of all laboratories	World average and standard deviation of grand-mean differences from NIST		World average and standard deviation of differences from world average of 1975 ETL intercomparison	
	Av unc^a^ (%)	Av^b^ (%)	Std dev^b^ (%)	Av (%)	Std dev (%)
250	2.82	1.10	0.88		
260	2.30	0.43	0.48		
270	1.75	0.92	2.54		
280	1.83	0.78	2.68		
290	1.69	0.55	2.44		
300	2.69	0.57	2.32	0.00	1.33
310	2.18	−0.38	1.95		
320	2.15	−0.37	2.22		
330	1.88	−0.38	2.09		
340	1.76	0.09	2.12		
350	1.66	0.66	1.32	0.00	1.14
400	1.31	0.85	0.95	0.00	1.42
450	1.26	0.89	0.96		
500	1.09	0.70	0.84	0.00	0.77
555	1.04	0.58	0.86		
600	1.05	0.50	0.74	0.00	0.54
654.6	1.07	0.12	0.61		
700	1.00	0.42	0.70	0.00	0.54
800	1.09	−0.29	0.84	0.00	2.42
900	1.31	−1.38	1.95		
1050	1.54	−0.78	2.44		
1150	1.54	−0.48	2.72		
1200	1.55	−0.21	2.63		
1300	1.59	0.17	2.57		
1540	1.44	0.73	3.05		
1600	1.57	−0.50	2.73		
1700	1.54	0.66	3.14		
2000	1.61	0.69	2.80		
2100	1.77	−1.14	3.03		
2300	1.87	−0.34	3.84		
2400	1.95	−0.83	3.66		

a Does not include the uncertainties of NIM from 250 to 280 nm or of NPL from 900–2400 nm.

b Does not include the grand-mean differences of NIM from 250 to 280 nm or of NPL from 900–2400 nm.

## Footnotes

1 Data submitted by the Amt fuer Standardisierung, Messwesen und Warenpruefung (ASMW) of the former German Democratic Republic were withdrawn after the unification of Germany.

2 Certain commercial equipment, instruments, or materials are identified in this paper to specify adequately the experimental procedure. Such identification does not imply recommendation or endorsement by the National Institute of Standards and Technology, nor does it imply that the materials or equipment identified are necessarily the best available for the purpose.

3 The 1.1 factor was applied to avoid computer rejection of data that fell just barely outside the one standard deviation level.

4 The world average and the standard deviation of differences from world average were recalculated without using the ASMW data.

## 8. Appendix A. Descriptions of the Scale Derivations of the Participating Laboratories

Each laboratory was asked to give a one page or less description of the derivation of its spectral irradiance scale. These descriptions that were submitted are given here.

### 8.1 Derivation of the NIST (USA) Spectral Irradiance Scale

#### Realize Spectral Radiance Scale

1. A beam conjoiner is used to measure the responsivity (linearity) of the photomultiplier amplifier system of our spectroradiometer.
2. Lamp V25 (high stability Quinn-Lee type vacuum lamp) is calibrated against a gold point blackbody at 654.6 nm.
3. Lamp C706 (high stability GEC type vacuum lamp) is calibrated against V25 at 654.6 nm—C706 has approximately eight times the output of V25 at 654.6 nm.
4. Lamp C706 is used to determine the temperature of a variable temperature blackbody which operates over a range of 1050 to 2700 K.
5. The spectral radiance of a test source is compared to the spectral radiance of the blackbody at some test wavelength and Placnk’s equation is used to determine the spectral radiance of the test source.

#### Realize Spectral Irradiance Scale on Primary Working Standards

1. Steps 4 and 5 from above are carried out to perform a spectral radiance calibration on the output of a small integrating sphere source (5.1 cm diameter sphere with a 1.8 cm diameter exit aperture) from 250 to 2400 nm.
2. Our spectroradiometer is then put in its spectral irradiance mode where it has a small averaging sphere (3.8 cm diameter sphere with a 1.2 cm entrance aperture) as the receiving optic.
3. At each wavelength the output of the primary working standards are compared to the output of the integrating sphere source.
4. The aperture sizes of the integrating sphere and the averaging sphere are accurately known and we accurately measure the distance between the two apertures. With this information we can calculate the spectral irradiance of the sphere source at the aperture of the averaging sphere.
5. After determining the ratio of a primary working standard output to the sphere source output, we can calculate the spectral irradiance of the primary working standard. This is done for each primary working standard.

#### Spectral Irradiance Scale Transfer

1. Four primary working standards are used to calibrate a group of 12 test lamps.
2. Each test lamp is measured four times-once in each of four test positions and against each of the four primary working standards.

### 8.2 Derivation of the CSIRO (Australia) Spectral Irradiance Scale

#### Relative Spectral Radiance Scale

The temperature of a tungsten-tube type cavity radiator (Osram lamp type Wi80) at about 2700 K was measured using broad-band filters, a non-selective thermal detector and application of Planck’s law. Using Planck’s law the relative spectral radiances were calculated for the limited wavelength range 350–800 nm.

#### Relative Spectral Irradiance Scale

1. A matched-pair of colour-corrected lenses, in conjugate positions with a magnification or de-magnification of a factor of ten, was used to image the selected region of the cavity lamp or the whole filament of an irradiance standard lamp (Wotan type Wi41G) in turn, within the entrance aperture of an integrating sphere attached to a spectroradiometer. The transfer was also limited to the wavelength range 350–800 nm.
2. The scale of relative spectral irradiance was transferred from the Wotan lamp standards to working standard tungsten-halogen lamps (Ushio Electric 100 V 500 W) by irradiating in turn a barium sulphate plate which was imaged onto the spectroradiometer entrance slit.

#### Spectral Irradiance Scale—SI Units

The illuminance of each working standard tungsten-halogen lamp was measured at the defined position using an electrical substitution absolute radiometer fitted with a V(λ) correction filter with accurately-measured spectral transmittances. The conversion factor 683 lm/W was used for the wavelength 555 nm, and correction was made for departure of the detector from V(λ). From the illuminance and the known relative spectral power distribution the spectral irradiances were calculated for the wavelength range 350–800 nm.

#### Spectral Irradiance Scale—uv and ir Spectral Regions

The CSIRO Spectral Irradiance Scale for the wavelength ranges 250–350 nm and 800–2400 nm was adopted in 1975 from the ETL (Japan) scale of 1973. We also observed at the time good agreement with the NPL (UK) scale over the range 290–760 nm. We have no knowledge of the dependence of this borrowed scale on IPTS scales.

As the CSIRO scale is not independently based for the wavelength ranges 250–340 nm and 850–2400 nm, we suggest that only values from the CSIRO scale for the wavelength range 350–800 nm be used for the calculation of international mean values.

### 8.3 Derivation of the ETL (Japan) Spectral Irradiance Scale

#### Realization of Spectral Radiance Scale on Shelf Standards—A

1. Three blackbodies, a gold-point, a nickel-tube, and a graphite-tube were constructed.
2. The temperature of the graphite-tube black-body, which operated over a range of 2200 to 2500 K, was determined from the measured ratio of its spectral radiance to that of the gold-point black-body at 550, 600, and 650 nm.
3. The temperature of the nickel-tube blackbody, which operated within a range of 1520 to 1570 K, was determined by optical pyrometer calibrated against NRLM standard of radiance temperature.
4. Spectral radiances of six tungsten strip lamps (GE 30A/T24) were calibrated against those of the blackbodies from 250 to 2500 nm; all of the three blackbodies were referenced for the wavelengths longer than 1200 nm, whereas only the graphite-tube one was referenced for the shorter wavelengths.

#### Realization of Spectral Irradiance Scale on Shelf Standards—B

1. Spectral distributions of radiation from three quartz-bromine lamps (Ushio JPD-100-500-CS) were measured by comparing spectral irradiances alternately produced on a white diffusing surface by each of the lamps and by one of the strip lamps (our shelf standards of spectral radiance); two strip lamps were used.
2. The absolute value of spectral irradiance of each of the quartz-bromine lamps was measured at 580 and 600 nm by comparing spectral radiance of a smoked MgO surface irradiated by the lamp with that of one of the strip lamps; two strip lamps were used; the spectral radiance factor of the smoked MgO surface was determined from measured reflectance.
3. The absolute value of integrated irradiance from each of the quartz-bromine lamps was measured by a radiometer combined with a band-pass (300–2750 nm) filter; the radiometer was calibrated in terms of the ETL absolute radiometric scale. The absolute value of spectral irradiance was calculated from the measured integrated irradiance, spectral distribution determined in step 1, and the spectral transmittance of the filter.
4. The absolute value of spectral irradiance at 580 and 600 nm was determined by averaging the results of steps 2 and 3.
5. The spectral irradiance at the whole wavelength range of 250 to 2500 nm was determined by combining the results of steps 1 and 4.

#### Amendment of Spectral Irradiance Scale in the Shorter Wavelengths

1. Spectral distribution of the shelf standards was compared with that of synchrotron radiation over the wavelength range of 250 to 600 nm.
2. The spectral irradiance scale determined in step 5 of part B was amended in order that the spectral distribution would conform with the result of step 1 for the wavelengths shorter than 500 nm.

### 8.4 Derivation of the INM (France) Spectral Irradiance Scale

#### Realize Spectral Radiance Scale

1. A separate apparatus is used to measure the linearity of photomultiplier and photodiode of our spectroradiometer.
2. Two lamps 337C and 340C (20–24G GEC type gas filled lamps) are calibrated against radiance temperature standards of our pyrometry laboratory (high stability GEC type vacuum lamp calibrated against gold point black body).
3. These lamps are used to determine the temperature of a variable temperature black body which operates over a range of 1500 to 1950 K.
4. The spectral radiance of test sources (339C and 367C) is compared to the spectral radiance of the black body and Planck’s equation is used to determine the spectral radiance of the test source.

#### Realize Spectral Irradiance Scale on Intercomparison Lamps

1. At each wavelength our spectroradiometer is switched from radiance mode for response to radiance standard, to irradiance mode for response to irradiance source.
2. Relative irradiance values are calculated from ratio of these two responses. Irradiance is fixed to 1 for 550 nm.
3. Luminous intensity of intercomparison lamps is compared to luminous intensity standards. Luminous intensity scale is based on use of electrical substitution pyroelectric radiometer.
4. From relative irradiance values and luminous intensity we can calculate irradiance values.
5. Each intercomparison lamp is compared to two radiance standards lamps and four luminous intensity lamps.

### 8.5 Derivation of the IOM (Spain) Spectral Irradiance Scale

The spectral irradiance scale of the Institute of Optics is based on an electrically calibrated pyroelectric radiometer and maintained in FEL type lamps.

The derivation process is as follows:

- Measurement of the spectral transmittance of interference filters.
- Measurement of the irradiance produced by the lamps, filtered by the interference filters, at 50 cm away from the lamp by using the ECPR.
- Corrections to the measurements in order to take into account filters thickness and bandwidth.
- Interpolation of the lamp spectral irradiance values from the measurements points. The De Voss’ approximation is used.

### 8.6 Derivation of the NIM (People’s Republic of China) Spectral Irradiance Scale

Primary standard is a high temperature (about 2800 K, variable) black body radiator. A precise circular diaphragm is placed at the exit port of the radiator. The spectral irradiance was measured at the place which is departure from the precise diaphragm in a fixed distance (500 mm).

The secondary standard is a group of 10 tungsten-halogen lamps (1000 W).

The comparator is a high accuracy wide wavelength range spectral radiometer which was developed at NIM.

### 8.7 Derivation of the NPL (UK) Spectral Irradiance Scale

The NPL spectral irradiance scale is based, over the wavelength range 350–800 nm, on comparisons with a black body cavity radiator. The spectral radiance of a group of tungsten ribbon filament lamps was measured by a direct comparison with the radiance of the cavity radiator and a radiance/irradiance transfer was then carried out to establish a relative spectral irradiance scale. The irradiance scale is held by a group of secondary standard coiled tungsten filament lamps.

In order to maintain internal consistency between the various optical radiation scales maintained at NPL, the absolute level of the scale has been assigned by relating it to the national scale of luminous intensity which was established radiometrically and is based on the NPL cryogenic radiometer.

At wavelengths from 200–370 nm, the relative spectral power scale has been established based on synchrotron radiation, which was used to calibrate a group of low pressure deuterium lamps. Again, the first stage of the calibration involved the establishment of a scale of spectral radiance which was then used to provide a scale of relative spectral irradiance on the same deuterium lamps. The absolute level has been assigned by comparison at 350 nm with the black body based scale described above.

The accuracy of the scale has subsequently been checked and confirmed at a number of points in over the wavelength range 400–800 nm by the use of a series of filter radiometers, each consisting of a specially constructed narrow band interference filter combined with a silicon call. The spectral responsivity curve for each of the radiometers was characterized using a tuneable dye laser and the absolute responsivity obtained by a direct comparison with the NPL spectral responsivity scale based on the cryogenic radiometer.

When the intercomparison started, work was in progress to extend the wavelength range covered by the NPL spectral irradiance scale into the infrared, but this work had not been completed. Rather than opt out completely in the infrared, NPL decided to measure the lamps and submit results based on a provisional calculation using published data for the emissivity of tungsten. It was hoped that these results could later be corrected to incorporate the new scale before the end of the intercomparison. Unfortunately, however, further unforseen problems were encountered which prevented NPL from completing the work on time.

### 8.8 Derivation of the DPT (South Africa) Spectral Irradiance Scale

Our spectral irradiance measurements were made over the wavelength region 400–1300 nm.

The method was based on an absolute radiometer and a series of interference filters which were used to determine the spectral irradiances of quartz halogen incandescent lamps at a number of discrete wavelengths. Values at intermediate wavelengths were obtained by interpolation.

The spectral transmittances of 11 interference filters together with a Schott K50 glass filter were measured with a Jobin Yvon Model HRD1 double monochromator, using a configuration which closely reproduces the actual geometry used in the spectral irradiance measurements. These measurements were verified on a Hitachi Model U-3400 spectrophotometer.

A pyroelectric detector, previously calibrated against an absolute radiometer, was then used in conjunction with the filter combinations mentioned above to measure the actual spectral irradiances around the different effective wavelengths.

Irradiance values at intermediate wavelengths were determined by a least square fitting to a polynomial function.

### 8.9 Derivation of the NRC (Canada) Spectral Irradiance Scale

#### UV-Visible (300–800 nm)

This scale is maintained on eight 500W quartz-halogen lamps similar to the lamps used in the last intercomparison [Suzuki and Ooba, Metrologia 12, 123 (1976)]. The working distance is 50 cm. The lamps were calibrated originally by means of an NBS spectral irradiance standard. After the above intercomparison, the spectral irradiance values of the eight lamps were adjusted to be equal to the world mean of that intercomparison. These adjusted values have been used since, without applying ageing corrections.

#### Near-Infrared (700–1600 nm)

This is a new scale, realized at NRC in 1989–90. Electrical substitution absolute radiometers were used in conjunction with interference filters to measure directly the spectral irradiance of FEL and NPL/GEC tungsten-halogen lamps from 700 nm to 1600 nm, at 100 nm intervals. The working distance is 100 cm. Interpolation techniques are used to obtain values at intermediate wavelengths.

#### For This Intercomparison

The old scale (300–800 nm) was used to calibrate the lamps from 300 nm up to and including 654.6 nm. The lamps used in the intercomparison are some of the lamps used to realize the new scale in the near ir. The new scale spectral irradiance values were used from 700 to 1600 nm. A correction factor was applied to transfer from a working distance of 100 cm to a working distance of 50 cm. This correction factor was determined for each lamp by measuring the variation of irradiance with distance.

### 8.10 Derivation of the OMH (Hungary) Spectral Irradiance Scale

#### Realization of Spectral Irradiance Scale

1. Today our spectral irradiance scale is based on two FEL 8A/1000 W irradiance standard lamps calibrated by NIST.
2. We have developed a wide band filter-radiometer with eight bandfilters made of absorbing glasses and a precision aperture. The spectral response calibration of the device is based on our absolute spectral responsivity scale and high accuracy spectral transmittance measurements.
3. The integrated spectral responses were measured for the investigated irradiance standard lamps by means of the eight bandfilters.
4. The spectral irradiance of the standard lamps in the spectral range of 330-800 nm is being determined from the eight measurement results with the help of a least squares fitting de-convolution program. This program needs further development.
5. The measurement results sent by OMH for the intercomparison are based on our calibrated lamps. We plan to send our bandfilter-radiometer based results too by May.

#### Spectral Irradiance Scale Transfer

1. Our spectral irradiance transfer spectroradiometer consists of a small averaging sphere (10 cm diameter sphere with a 2.6 cm entrance aperture) as a receiving optic, a double grating monochromator, imaging optics, detectors (Si photodiode, PbS photoresistor, photomulti-plier) and an ac current to voltage converter lock in system.
2. At each wavelength the output of our spectral irradiance standards were measured with our spectroradiometer.
3. At each wavelength the output of the test lamps were measured with our spectroradiometer.
4. Then the standard lamps were remeasured at each wavelength in order to take in consideration the long term instability of the measuring system.
5. After determining the ratio of the standard lamps output to the test lamps output, we can calculate the spectral irradiance of the test lamps.
6. Each lamp was measured four times.

### 8.11 Derivation of the PTB (Germany) Spectral Irradiance Scale

The spectral irradiance scale is based on a Planckian radiator with accurately known temperature and area realized by a variable-temperature blackbody with a large-area, water-cooled measuring aperture. The blackbody temperature of approximately 2800 K is measured with a linear pyrometer calibrated at PTB. The spectral irradiance at an accurately measured distance of 1 m to the aperture is derived from the spectral radiance of the Planckian radiator at the set temperature and the accurately measured area of the aperture with a diameter of approximately 10 mm. The transfer of the spectral irradiance scale of the blackbody to that of the standard lamp(s) is per^ formed by comparing the radiance of a plane reflection standard (BaS04) defining the measuring plane which is perpendicularly irradiated by both sources, blackbody and standard lamp(s), in succession. Each of our three CCPR test lamps is calibrated three times per round at a distance of 70 cm to the measuring plane against a group of four standard lamps where all lamps are placed on the same optical axis. In addition, an uncalibrated highly stabilized monitor or comparison lamp is always used to correct for residual instabilities of the electrical and optical setup during the measurement. The factors to correct the spectral irradiance of the test lamps for the required distance of 50 cm are measured separately, where it is verified that the factors are independent of wavelength.

An improved spectroradiometer is just being put into operation.

### 8.12 Derivation of the VNIIOFI (USSR) Spectral Irradiance Scale

#### Lamp Calibration Method

Calibrations of the lamps have been carried out against the Radiometric Standard of the USSR. Method of measurements is based on the use of a graphite blackbody model (BBM) of BBM-2500 type. Operating temperatures of the BBM can vary within the range of 1700 to 2600 K. The BBM temperature was maintained with the accuracy of ± 0.3 K. BBM-2500 was installed inside a vacuum chamber provided with a quartz output window which transmission spectrum was measured by the standard spectrophotometer.

BBM was mounted on a special support together with a test lamp, a set of screens, a rotatable integrating sphere and a mirror condenser used to focus the integrating sphere exit port onto the spectrometer entrance slit. The integrating sphere is made of “Halon” type material and has the following dimensions: the sphere diameter—40 mm, the entrance and exit port diameters —11 mm and 5 mm, respectively, with the angle between the ports being 90°. The sphere is fitted with a rotary mechanism to allow for turning the entrance port to face either the BBM or the test lamp. The condenser is built of two off-axis parabolic mirrors with a focus distance of 822 mm. The double monochromator HRD-1 (“Jobin Yvon,” France) in combination with a set of photomultipliers and photoresistance cells was used as a spectrometer.

Calibration of the test lamps for spectral irradiance consisted of two stages.

1. During the first stage, the relative spectral irradiance distribution of the lamps was measured. Incidentally, the distance from the entrance port of the integrating sphere to the lamp was set at 1200 mm, while that to the BBM aperture diaphragm was 300 mm. The BBM aperture diaphragm was placed inside a vacuum chamber and had a diameter of 8 mm. this provided for ensuring an adequate level of signal from BBM throughout the entire spectrum range. Independence of the relative spectral distribution of irradiance against a distance to a lamp and the BBM aperture diaphragm was under control.
2. During the second stage, the lamp and the BBM aperture diaphragm were positioned at a distance of 500 ±0.1 mm from the entrance port of the integrating sphere (besides, BBM aperture diaphragm was located outside the vacuum chamber and its diameter was 3 ± 0.001 mm) and the absolute spectrum of the lamp was found at several wavelengths in the visible region of the spectrum.

## 9. Appendix B. Plots Showing the Data Analysis for One of the Laboratories

Section 6 of the main paper describes the data analysis. A sample of the data analysis is given here. The data analysis for PTB is shown graphically in Figs. 6 to 10.

## 10. Appendix C. Plots of the Grand-Mean Percent Difference from NIST and the Combined Lab/NIST Uncertainty

Figures 11 to 21 show the grand-mean percent difference from NIST and the combined Lab/NIST uncertainty for each laboratory. The combined Lab/NIST uncertainty is the quadrature sum of the one standard deviation uncertainty reported by the laboratory and the one standard deviation uncertainty reported by NIST (given in Table 2).
